# Multiorgan Failure and Refractory Lactic Acidosis due to *Pasteurella multocida* Septicemia in a Patient with No Animal Exposure

**DOI:** 10.1155/2018/2574184

**Published:** 2018-03-22

**Authors:** Damaris Pena, Yaneidy Santana, Jose Perez Lara, Efrain Gonzalez, Misbahuddin Khaja

**Affiliations:** ^1^Division of Pulmonary and Critical Care Medicine, Bronx Lebanon Hospital Center Affiliated with Icahn School of Medicine at Mount Sinai, 1650 Grand Concourse, Bronx, NY 10457, USA; ^2^Department of Medicine, Bronx Lebanon Hospital Center Affiliated with Icahn School of Medicine at Mount Sinai, 1650 Grand Concourse, Bronx, NY 10457, USA; ^3^Division of Infectious Disease Medicine, Bronx Lebanon Hospital Center Affiliated with Icahn School of Medicine at Mount Sinai, 1650 Grand Concourse, Bronx, NY 10457, USA

## Abstract

**Introduction:**

*Pasteurella multocida* is a gram-negative coccobacillus pathogenic to animals. It can cause infection in humans by a bite, scratch, or lick from a cat or dog. *P. multocida* can cause a variety of infections in humans, including cellulitis, osteomyelitis, endocarditis, peritonitis, and septic shock.

**Case Presentation:**

A 56-year-old male presented to our hospital with a 2-day history of fever, abdominal pain, nausea, and vomiting. He denied exposure to cats, dogs or other pets. He had severe respiratory distress requiring ventilator support, profound septic shock requiring multiple vasopressors, severe lactic acidosis, and renal failure requiring emergent hemodialysis. Blood cultures confirmed the presence of *P. multocida*. The patient subsequently died of cardiopulmonary arrest due to multiorgan failure with refractory shock.

**Conclusion:**

*P. multocida* septicemia can lead to septic shock. Early identification of this organism may decrease mortality. Although our patient had no known cat or dog exposure, physicians should enquire about a history of animal exposure when a patient presents with an infection with no obvious cause.

## 1. Introduction

*Pasteurella multocida* is a gram-negative coccobacillus present in the respiratory tract of dogs, cats, other felines, and fowl [1]. The *Pasteurella* genus contains a variety of species, including *P. multocida*, *P. gallicida*, *P. canis*, *P. dagmatis*, *P. septica*, and *P. stomatis* [2]. Human infections by *P. multocida* most commonly occur after receiving a scratch, lick, or bite from a cat or dog. There are also case reports of *P. multocida* infection in healthy individuals in the absence of dog or cat exposure [3].

On average, animal bites account for more than 300,000 emergency room visits annually in the United States. With bacteremia caused by *P. multocida*, mortality rates ranged from 7% to 31% [4]. In the absence of an animal bite, the mortality rate of *P. multocida* infection was 21% [5].

Herein, we describe a man with *P. multocida* septicemia, which is rare because of its occurrence in the setting of no apparent exposure to dogs, cats, or other animals. The infection progressed rapidly, leading to refractory lactic acidosis with multiorgan failure and treatment-resistant shock.

## 2. Case Presentation

A 56-year-old Hispanic male with no comorbid conditions presented to our emergency department with a 2-day history of fever, generalized weakness, abdominal pain, nausea, and vomiting. He had no hemoptysis, hematemesis, arthralgia, or headache. He denied recent contact with sick humans, exposure to pets or birds, or travel. He smoked 5 cigarettes per day for over 20 years but denied using alcohol or recreational drugs.

On physical examination, the patient was in respiratory distress, with a respiratory rate of 24 breaths per minute and pulse oxygen saturation of 90% on 2 liters per minute oxygen via a nasal cannula. His temperature was 101.5°F, heart rate was 68 beats per minute, and blood pressure was 80/50 mm·Hg. On lung examination, bilateral rales were noted. His heart sounds were normal. His abdomen was soft upon palpation, with slight tenderness in the right upper quadrant; no organomegaly was noted. Neurologic examination was unremarkable. The patient was intubated immediately because of his respiratory distress, and an intravenous infusion of norepinephrine was begun for the presumptive diagnosis of septic shock.

**Figure 1:**
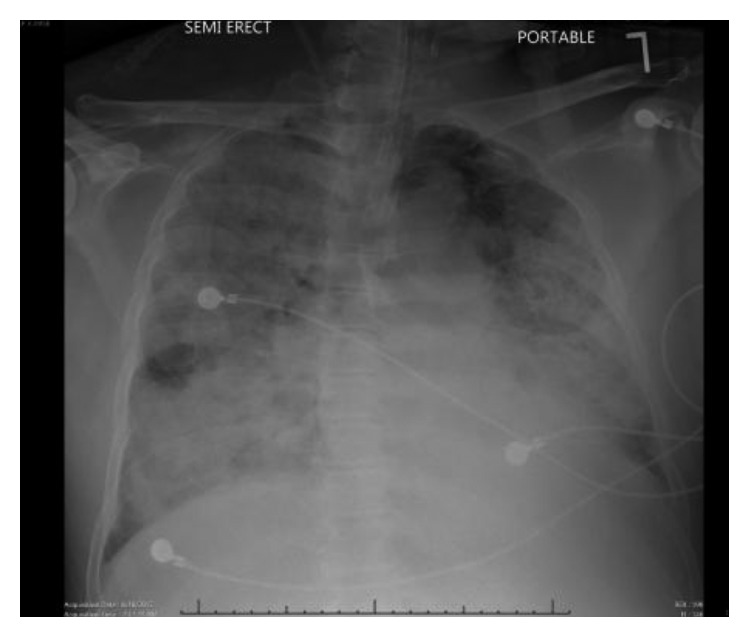
Chest X-ray showing diffuse bilateral alveolar infiltrates.

Relevant laboratory results on the day of admission were as follows: leukocytosis (white blood cell count: 20 × 10^3^ cells/*µ*L), lactic acidosis (serum lactate: 17.5 mmol/L), pH of 7.15, bicarbonate of 15 mEq/L, blood urea nitrogen of 37 mg/dL, serum creatinine of 4.5 mg/dL, and increased serum aspartate transaminase (1532 unit/L), alanine aminotransferase (683 unit/L), and alkaline phosphatase (149 unit/L). Chest X-ray showed bilateral diffuse alveolar infiltrates (Figure 1).

The patient underwent fiberoptic bronchoscopy with bronchoalveolar lavage; diffuse alveolar hemorrhage was excluded, and cultures from the lavage samples were negative. Bedside ultrasound of the abdomen revealed acalculous acute cholecystitis, and a percutaneous cholecystostomy tube was placed. Blood cultures from admission grew *P. multocida*, and gram-negative *Pasteurella* rods were seen on gram stain during examination via light microscopy. Cultures from the gallbladder fluid were negative. Transthoracic echocardiography showed a left ventricular ejection fraction of 74% and no vegetations.

The patient was diagnosed with multiorgan failure, septic shock with severe lactic acidosis, acute respiratory distress syndrome (ARDS), and renal failure requiring hemodialysis. Broad-spectrum intravenous antibiotics were begun. His condition rapidly deteriorated, and his clinical course was further complicated by disseminated intravascular coagulation, gastrointestinal bleeding, and refractory shock requiring multiple vasopressor medications. He subsequently died of a cardiopulmonary arrest. Despite all measures, the serum lactic acid at the time of his death was 12 mmol/L.

## 3. Discussion

*P. multocida*, which belongs to the Pasteurellaceae family, is a nonmotile gram-negative coccobacillus that is penicillin sensitive [6]. In 1878, *P. multocida* was first detected in cholera-infected birds. The organism was subsequently isolated by Louis Pasteur in 1880. *P. multocida* infections in humans can occur by contact with dogs, cats, and other felines [7]. The primary mechanism of transmission of *P. multocida* to humans is by direct or indirect contact with animals. Rarely, human-to-human and vertical transmission have been reported [8].

*P. multocida* has a polysaccharide capsule and a lipopolysaccharide surface molecule that allow it to resist phagocytosis by host cells and complement-mediated lysis [9]. Immunocompromised humans are particularly susceptible to developing an infection from *P. multocida*. These include individuals with cirrhosis, diabetes, malignancies, chronic obstructive pulmonary disease, and kidney failure requiring dialysis.

A study done by Vondra and Myres reported septic shock and acute sepsis syndrome in more than one-third of patients. *P. multocida* bacteremia had a mortality rate of 22.6%. In this study, most of the patients had a significant underlying medical illness, and the patients who died were immunocompromised [10]. The other clinical manifestations of *P. multocida* include skin and soft tissue infections, respiratory tract infections, pneumonia, intra-abdominal pelvic infections, spontaneous bacterial peritonitis, tubo-ovarian abscess endometriosis, pyogenic arthritis, endocarditis, osteomyelitis, genitourinary tract infections, pyelonephritis, cystitis. Rare manifestations include uvulitis and pharyngitis. Central nervous system manifestations such as meningitis, subdural empyema and ocular infections have been reported [10, 11]. Our patient did not have any prior medical illnesses on presentation and was not immunocompromised. Our patient did not have any exposure to felines.

Upper respiratory tract infections caused by *P. multocida* include pharyngitis, sinusitis, otitis media, epiglottitis, and rarely Ludwig's angina [12]. Lower respiratory tract infections caused by the bacteria include tracheobronchitis, pneumonia, empyema, and abscess. Multilobar and diffuse infiltrates have been previously reported [13].

Sepsis and septic shock have been reported with *P. multocida* [14], as well as peritonitis in patients receiving peritoneal dialysis [15]. Michel et al. reported a case of a patient who underwent thoracoabdominal esophagectomy and developed acute respiratory distress syndrome (ARDS) postoperatively, whose protected specimen brush sample showed *P. multocida* [16]. Christidou et al. reported in their study that two patients died after developing acute respiratory syndrome (ARDS) from *P. multocida* pneumonia. The third patient who initially presented with intracranial hemorrhage requiring craniotomy also died of *P. multocida* pneumonia [17].

A case similar to ours was presented by Arora et al. where a patient rapidly progressed to severe sepsis syndrome with acute kidney injury, acute respiratory distress syndrome (ARDS) and multiorgan dysfunction syndrome. Their patient had an immunocompromising condition, lupus nephritis, and exposure to puppies at home. Our patient was not immunocompromised and did not have exposure to felines [18]. Nagata et al. described a patient with cholecystitis caused by *P. multocida*; our case presented with similar findings [19]. This patient, like ours, had a high leukocyte count (due to the severe inflammatory reaction caused by infection with this organism), with a white blood cell count of 47 × 10^3^ cells/*µ*L on the day of admission.

Depending on the site of infection, *P. multocida* organisms may be isolated from wound, sputum, blood culture, bronchoalveolar lavage, pleural fluid, ascitic fluid, or cerebrospinal fluid. In our patient, the organism was isolated from the blood. *P. multocida* organisms grow on blood or chocolate agar at 37°C and produce a characteristic mousy odor [20, 21]. As the organism is susceptible to penicillin, this is the first-line antibiotic. Second- and third-generation cephalosporins, tetracycline, and carbapenems are drugs of choice for patients allergic to penicillin [22, 23].

Our patient had refractory lactic acidosis secondary to septic shock from underlying *P. multocida* infection. Lactic acidosis is caused by decreased tissue oxygenation from impaired blood flow or underlying medical conditions, medications, intoxications, and inborn errors of metabolism leading to lactate accumulation in body. Overproduction or decreased utilization of lactate leads to lactic acidosis. Refractory lactic acidosis can be caused by various conditions including low hemoglobin with low oxygen-carrying capacity, decreased delivery of oxygen due to circulatory problems and low partial pressure of oxygen due to lung disease [24].

Our case is unusual, as the patient developed multiorgan failure with severe lactic acidosis caused by *P. multocida* septicemia. He had renal failure requiring hemodialysis, severe ARDS with a PaO_2_/FiO_2_ ratio less than 100, septic shock requiring multiple vasopressors, and fulminant liver failure.

## 4. Conclusion

When a patient presents with sepsis or other infection with no obvious cause, a history of pet exposure, whether recreational or occupational, should be solicited by the physician. If positive, this should lead to a high clinical suspicion for *P. multocida* infection, although not all patients infected with the organism will have a positive history. Early identification of *P. multocida* infection may decrease mortality. Person-to-person transmission is uncommon, but maintenance of hand hygiene may decrease the spread of this infection.

## Abbreviations

*P. multocida*: *Pasteurella multocida*
ARDS: Acute respiratory distress syndrome.

## References

[1] Khan M. F., Movahed M. R., Jung J. (2012). *Pasteurella multocida* endocarditis. *Journal of Heart Valve Disease*.

[2] Donnio P. Y., Lerestif-Gautier A. L., Avril J. L. (2004). Characterization of *Pasteurella* spp. strains isolated from human infections. *Journal of Comparative Pathology*.

[3] Ruiz-Irastorza G., Garea C., Alonso J. J. (1995). Septic shock due to *Pasteurella multocida* subspecies multocida in a previously healthy woman. *Clinical Infectious Diseases*.

[4] Narsana N., Farhat F. (2015). Septic shock due to *Pasteurella multocida* bacteremia: a case report. *Journal of Medical Case Reports*.

[5] Giordano A., Dincman T., Clyburn B. E. (2015). Clinical features and outcomes of *Pasteurella multocida* infection. *Medicine*.

[6] Kuhnert P., Christensen H. (2008). *Pasteurellaceae: Biology, Genomics and Molecular Aspects*.

[7] Klein N. C., Cunha B. A. (1997). *Pasteurella multocida* pneumonia. *Seminars in Respiratory Infections*.

[8] Nakwan N., Nakwan N., Atta T., Chokephaibulkit K. (2009). Neonatal pasteurellosis: a review of reported cases. *Archives of Disease in Childhood-Fetal and Neonatal Edition*.

[9] Chung J. Y., Wilkie I., Boyce J. D. (2001). Roleof capsule in the pathogenesis of fowl cholera caused by *Pasteurella multocida* serogroup A. *Infection and Immunity*.

[10] Vondra M. S., Myres J. P. (2011). *Pasteurella multocida* bacteremia: report of 12 cases in the 21st century and comprehensive review of adult literature. *Infectious Diseases in Clinical Practice*.

[11] Hazouard E., Ferrandière M., Lanotte P. (2000). Septic shock caused by *Pasteurella multocida* in alcoholic patients. Probablecontamination of leg ulcers by the saliva of the domestic cats. *Presse Medicale*.

[12] Harris P. J., Osswald M. B. (2010). *Pasteurella multocida* epiglottitis: a review and report of a new case with associated chronic lymphocytic leukemia. *Ear, Nose, and Throat Journal*.

[13] Kopita J. M., Handshoe D. (1993). Cat germs! Pleuropulmonary *Pasteurella* infection in an old man. *North Carolina Medical Journal*.

[14] Kimura R., Hayashi Y., Takeuchi T. (2004). *Pasteurella multocida* septicemia caused by close contact with a domestic cat: case report and literature review. *Journal of Infection and Chemotherapy*.

[15] Malik A., Al Aly Z., Mailey K. S., Bastani B. (2005). *Pasteurella multocida* peritoneal dialysis-associated peritonitis: a report of two cases and review of the literature. *Journal of Nephrology*.

[16] Michel F., Allaouchiche B., Chassard D. (1999). Postoperative adult respiratory distress syndrome (ARDS) due to *Pasteurella multocida*. *Journal of Infection*.

[17] Christidou A., Maraki S., Gitti Z., Tselentis Y. (2005). Review of *Pasteurella multocida* infections over a twelve-year period in a tertiary care hospital. *American Journal of Infectious Diseases*.

[18] Arora A., Payan H., Levine S. (2012). Dogs-man’s best friend? a case of *Pasteurella multocida* bacteremia with MODS. *Chest*.

[19] Nagata H., Yamada S., Uramaru K., Kiyasu Y., Kano N. (2014). Acute cholecystitis with bacteremia caused by *Pasteurella multocida*. *Surgical Infections*.

[20] Ebright J., Frey A., Fairfax M. (2009). *Pasteurella multocida* infections and bacteremia. *Infectious Diseases in Clinical Practice*.

[21] Escande F., Lion C. (1993). Epidemiology of human infections by *Pasteurella* and related groups in France. *Zentralblatt Für Bakteriologie*.

[22] Lion C., Conroy M. C., Carpentier A. M. (2006). Antimicrobial susceptibilities of *Pasteurella* strains isolated from humans. *International Journal of Antimicrobial Agents*.

[23] Goldstein E. J., Citron D. M., Merriam C. V. (2001). Comparative in vitro activity of ertapenem and 11 other antimicrobial agents against aerobic and anaerobic pathogens isolated from skin and soft tissue animaland human bite wound infections. *Journal of Antimicrobial Chemotherapy*.

[24] Kraut J. A., Madias N. E. (2014). Lactic acidosis. *New England Journal of Medicine*.

